# A Comprehensive Evaluation of Food Security in China and Its Obstacle Factors

**DOI:** 10.3390/ijerph20010451

**Published:** 2022-12-27

**Authors:** Yan Zhang, Xiaoyong Lu

**Affiliations:** 1 School of Public Policy and Administration, Nanchang University, Nanchang 330031, China; 2 Gongqing Institute of Science and Technology, Jiujiang 332020, China

**Keywords:** food security, comprehensive evaluation, obstacle factors, matter–element extension model, China

## Abstract

China’s food security has attracted global attention as the various drivers of its instability and uncertainty have intensified. This study developed a new framework for food security evaluation in China by analyzing its availability, distribution, utilization, vulnerability, sustainability, and regulation. The entropy weight method (EWM) and the matter–element extension model (MEEM) were combined to examine China’s food security status between 2001 and 2020. Additionally, an obstacle degree model (ODM) was used to investigate the key factors functioning as obstacles to food security. The results show that China’s overall food security improved greatly but experienced a slight downward trend in 2003. The main obstacles initially entailed grain distribution but then spread to vulnerability- and sustainability-related issues. Ultimately, the key factors restricting China’s food security were the amount of fertilizer application per unit sown area (AFA) and the grain self-sufficiency rate (GSR). The next 40 years could be the most critical period for ensuring China’s food security, which incorporates demographic, climate change, and resource shortage factors. China appears to be implementing its national strategies through sustainable farmland use and agricultural technology innovation to facilitate the high-quality development of its grain industries and strengthen its food security. This study provides an overall picture of China’s food security and can serve as a reference for those concerned with China’s future national security.

## 1. Introduction

As food is a key determinant of national prosperity and human wellbeing, its security represents a major prerequisite for national security [1]. In recent years, food security has been a concern in both developing and developed regions of the world [2], but the situation is particularly alarming in Asia and Africa, where the number of people experiencing hunger reached 418 million and 282 million in 2020, respectively [3,4]. China, home to one-fifth of the world’s population, faces different degrees of food production pressures. To maintain food security and to meet the demand of a large population, more grain needs to be produced on the 9% of global arable land and using 6% of global water resources, which suggests that future environmental and resource challenges will increase [5]. Further complicating this situation is that climate change, urbanization, and a shift in food habits from cereals to more meat products have caused or will cause great changes in food security in China [6]. Hence, a multidimensional measurement of China’s food security situation will support a more comprehensive and objective assessment of China’s food security situation; quantitative and qualitative evaluations of the obstacles affecting China’s food security status play a crucial role in determining the central goals of China’s food security strategy and agricultural policy.

Food security has emerged as a concern for governments and academia over the past few decades. At the World Food Conference of 1974 in Rome, Italy, food security was defined for the first time as ‘ensuring that all people at all times have access to enough food for survival and health’ [7]. The concept of food security was refined in the 1996 World Food Summit Plan of Action, and ‘when all people, at all times, have physical and economic access to sufficient, safe and nutritious food to meet their dietary needs and food preferences for an active and healthy life’ was the most widely accepted. Since its proposal, the concept of food security has been researched abroad [8]. Some studies have concluded that it focuses on food availability, utilization, and sustainability, while others have characterized it as the eradication of poverty, malnutrition, and hunger [9,10]. Currently, most studies do not adequately consider the potential tension between food sustainability and the other dimensions of food security. In addition, little related research has addressed the application of policies. Thus, in this study, food security is described as a ‘state in which a region or nation can meet the sustainable and ecological standards, and provide people with sufficient nutrition and healthy food that conforms to certain cultural habits and preferences under the government’s macrocontrol and market regulation mechanism’.

Much effort has been made to understand food security in China. In 1995, Lester R. Brown published the book *Who Will Feed China?: Wake-Up Call for a Small Planet* [11], which sparked worldwide concern about China’s food security. However, Brown seems to have sounded a false alarm. Over recent years, China has been self-reliant in securing its own food supply, and its people have not only enough to eat but also a greater range of choices than previously. Notably, we realize that large populations coupled with an increasing intensity of extreme environmental events, i.e., floods, droughts, and extreme variability in temperature and rainfall, undoubtedly pose a grim challenge to the current food security in China [12,13]. Cultivated land is an essential resource and condition for grain production and the material basis for ensuring the effective supply of grain and other important agricultural products [14]. We should never ignore that due to unsustainable agricultural practices or agricultural land becoming urbanized or lost through, for example, desertification, salinization, or erosion [15]. Therefore, determining how to secure the supply of agricultural products and improve the sustainability of agricultural development under the constraints of limited resources and the need for environmental sustainability is the most important food security challenge that must be overcome [16,17,18]. Moreover, human factors, such as agricultural technology innovation, policy mechanism guarantees, and increases in farmland water-conservation-construction investment, can somewhat mitigate all types of negative impacts [19].

To identify the obstacles restricting food security, much effort has been made to evaluate food security. Several scholars have tried to use a single index, such as the proportion of undernourished individuals in a population [20], average dietary energy supply [21], food self-sufficiency rate [22], and prediction of grain supply and demand [23,24], to describe and evaluate it. Notably, these methods are not appropriate for conducting a specific and comprehensive evaluation. With the continuous enrichment and systematization of the concept of food security, many scholars have applied the comprehensive management evaluation method to describe it. Examples include gray relation analysis (GRA) [25], fuzzy comprehensive evaluation (FCE) [26], artificial neural networks (ANNs) [27], and data envelopment analysis (DEA) [28,29]. Through statistical analysis and calculation, GRA can deal with gray systems with partly clear and partly unclear information based on the correlation degree of factors; however, due to the lack of systematic analyses on the causal relationships between different factors, an objective portrayal of the real food security situation using the conventional tendency method is difficult to achieve. FCE solves the problem of the decision-making objective being fuzzy and difficult to quantify, by introducing the membership degree, but it cannot solve the problem of information duplication caused by the correlation between evaluation indicators. As a deep learning method, ANNs have an adaptive ability and fault tolerance in terms of nonlinear complex systems, but their accuracy is not high due to fewer training samples in practical applications when utilizing this technique. The DEA method evaluates the relative effectiveness of the same type of units according to multi-index input and output based on relative efficiency. However, it only shows the relative development indicators of the evaluation unit and cannot show the actual development level.

Among the various methods developed to solve real-world decision problems, matter–element extension analysis has been widely used in research, such as environmental quality assessment [30], land suitability evaluation [31], and risk assessment in urban network planning [32], but its application in evaluating food security is limited. Compared with traditional methods, the matter–element extension model (MEEM) can address contradictions qualitatively and quantitatively [33]. Moreover, He et al. [32] and Ye [34] found that it is more credible and practical. Therefore, to overcome the abovementioned deficiencies, using both entropy theory and MEEM represents a new approach. The entropy weight method (EWM) is used to determine the weight coefficient of each factor in an analysis, preventing subjectivity in the process of weight determination. Matter–element extension theory is introduced in this paper to determine the membership degree between index and grade using the relation function, thus addressing the problem of fuzzy correspondence between the evaluation index and grade.

The overall goals of this paper are to systematically evaluate food security status, explore the dominant obstacle indicators of China’s food security, and provide policy implications for a path forward. The remainder of the paper is organized as follows. Section 2 introduces the data sources, core model, and research methods. Section 3 discusses the results of the empirical study. Section 4 discusses the results and limitations. The final section summarizes the paper’s conclusions.

## 2. Materials and Methods

### 2.1. Evaluation Index System

Since we recognize that designing an evaluation index system is key to the evaluation process, our study concentrates on establishing a reasonable and objective food security evaluation index system. Based on a review of previous studies, this paper considers overall planning for grain availability and grain distribution, fully recognizes the important role of the vulnerability and sustainability of grain production and the effective and economical utilization of resources involved in ensuring food security, and closely integrates government regulation with improving food security. Based on the preceding analysis, an evaluation index system composed of a total of 22 evaluation factors for food security in China is established (Table 1); the system is divided into six major components: availability, distribution, utilization, vulnerability, sustainability, and regulation. Additionally, the selected indicators are in accordance with established principles of integrity, comparability, dynamics, and practicability.

### 2.2. Data Sources

According to China’s statistical information, grain output covers cereals, beans, and tubers by type of crop. The statistics for total grain production (TGP), output of major farm products, grain imports and exports, planting structure of grain crops (PSG), per capita disposable income of residents (pDIR), affected area of grain crops (AAG), per capita level of government expenditure for supporting local production (pSRP), and proportion of government expenditure for supporting agriculture to agricultural output value (PAGE) were sourced from the Statistical Yearbook published by the National Bureau of Statistics of China and the *China Rural Statistical Yearbook* for various years from 1991 to 2020. The historical annual data used in this paper were collected from the *Achievements of New China’s Economic and Social Development in 70 Brilliant Years (1949–2019)*, which includes the length of highways and railways, the amount of agricultural fertilizer application, the sown area of grain crops and the effective irrigation area.

The poverty incidence was derived from the *Poverty Monitoring Report of Rural, China*. The grain price volatility index was obtained from the annual data published in the *China Urban Life and Price Yearbook*. The data on the prevalence of undernourishment (3-year average) and cereal stock variation came from FAOSTAT Data of the FAO (United Nations). Natural disaster statistics were downloaded from the Emergency Events Database (EM-DAT) of the Center for Research on the Epidemiology of Disasters (CRED) (http://www.emdat.be/ accessed on 9 March 2021). Missing data were supplemented through the interpolation method.

### 2.3. Methods

#### 2.3.1. Matter–Element Extension Model

Cai [33] first proposed the MEEM, which is mainly used to conduct comprehensive multifactor evaluation involving complex contradictions. Based on the definition of each index state evaluation grade, the MEEM can obtain more comprehensive and rich evaluation information by selecting the interval division of the index and analyzing the correlation degree of each influencing factor according to the subordinate relationship of the data, which reflects the change trend of the safety grade among the indexes.

The MEEM assumes that matter, denoted by N, has a characteristic represented by c and a value for the characteristic denoted by v [33]. In this paper, the ordered triad composed of the matter (N), characteristic (c), and value of the characteristic (v) can be represented by R, which constitutes the basic matter–element of food security evaluation. The R matter–element has *n*-dimensional characteristics, and the matter–element is as follows:

(1) $$R=\left(N,c,v\right)=\left(\begin{array}{ccc} N & c_{1} & v_{1}\\ & c_{2} & v_{2}\\ & \vdots & \vdots \\ & c_{n} & v_{n} \end{array}\right)\left(i=1,2,\cdots ,n\right)$$

To effectively evaluate China’s food security, the evaluation results of food security are divided into five grades: I (insecurity), II (critical security), III (basic security), IV (comparative security), and V (security). According to the overall value range of the grading standard of the food security evaluation index, the node domain ($R_{p}$) is determined. The classical domain ($R_{o}$) of food security evaluation of grades I–V is divided by the equipartition method. Additionally, the evaluation index set $c=\left\{c_{1},c_{2},\cdots ,c_{21},c_{22}\right\}$ consists of 22 specific evaluation indicators.

(2) $$R_{p}=\left(N,c,v_{p}\right)=\left(\begin{array}{ccc} N & c_{1} & v_{p1}\\ & c_{2} & v_{p2}\\ & \vdots & \vdots \\ & c_{n} & v_{pn} \end{array}\right)=\left(\begin{array}{ccc} N & c_{1} & \left(a_{p1},b_{p1}\right)\\ & c_{2} & \left(a_{p2},b_{p2}\right)\\ & \vdots & \vdots \\ & c_{n} & \left(a_{pn},b_{pn}\right) \end{array}\right)$$

(3) $$R_{o}=\left(N,c,v_{o}\right)=\left(\begin{array}{ccc} N & c_{1} & v_{o1}\\ & c_{2} & v_{o2}\\ & \vdots & \vdots \\ & c_{n} & v_{on} \end{array}\right)=\left(\begin{array}{ccc} N & c_{1} & \left(a_{o1},b_{o1}\right)\\ & c_{2} & \left(a_{o2},b_{o2}\right)\\ & \vdots & \vdots \\ & c_{n} & \left(a_{on},b_{on}\right) \end{array}\right)$$

where N = {I, II, III, IV, and V} represents all the assessment grades. $v_{pi}$ is the range of characteristics $c_{i}$, denoted as the node domain of N, and $v_{oi}=\left(a_{oi},b_{oi}\right)\left(i=1,2,\cdots ,n\right)$ represent the value range of the grade k (k = I, II,…, V) in the matter–element system, that is, the classical domain taken by the evaluation index of each grade on the object, corresponding to the variation range of the value of each single-factor parameter in a certain category.

Based on the node domain and classical domain introduced above, a correlation function can be established to quantify the correlation degree $K_{i}\left(v_{i}\right)$.

(4) $$K_{i}\left(v_{i}\right)=\left\{\begin{array}{c} \frac{-d_{1}}{\left|v_{oi}\right|},v_{i}\in v_{oi}\\ \frac{d_{2}}{d_{2}-d_{1}},v_{i}\notin v_{oi} \end{array}\right.$$

(5) $$d_{1}=\left|v-\frac{1}{2}\left(a_{oi}+b_{oi}\right)\right|-\frac{1}{2}\left(b_{oi}-a_{oi}\right)\left(i=1,2,\cdots ,n\right)$$

(6) $$d_{2}=\left|v-\frac{1}{2}\left(a_{pi}+b_{pi}\right)\right|-\frac{1}{2}\left(b_{pi}-a_{pi}\right)\left(i=1,2,\cdots ,n\right)$$

Here, $d_{1}$ and $d_{2}$ represent the distances between $v_{i}$ and the classical domain $v_{oi}=\left(a_{oi},b_{oi}\right)\left(i=1,2,\cdots ,n\right)$ and the node domain $v_{pi}=\left(a_{pi},b_{pi}\right)\left(i=1,2,\cdots ,n\right)$, respectively. The variable $\left|v_{oi}\right|$ is the module of the bounded interval of the upper limit and lower limit of grade k for parameter i. Based on the weights from the EWM, the weighted correlation degree $K\left(R_{o}\right)$ is calculated using formula (7).

(7) $$K\left(R_{o}\right)=\overset{n}{\underset{i=1}{\sum }}wK_{i}\left(v_{i}\right)$$

In this paper, w is the weight of each index c obtained by the EWM. $K\left(R_{o}\right)$ expresses the correlation between the evaluation sample $R_{o}$ and its corresponding grade. According to the maximum recognition principle of the correlation degree, $K\left(t\right)$ indicates that the evaluation object $R_{o}$ belongs to grade k.

(8) $$K\left(t\right)=MAX\;K_{k}\left(R_{o}\right)$$

#### 2.3.2. Obstacle Degree Model

The obstacle degree model (ODM) can identify and analyze influencing factors, determine the key factors that have a major impact on the evaluation results, clarify the impact degree of key constraints, and help achieve sustainable development of grain output and better ensure China’s food security [35,36,37]. Therefore, this paper introduces index contribution $w_{j}$, indicator deviation $T_{ij}$, and obstacle degree $O_{j}$ to identify the obstacle degree of the factors affecting food security. The calculation method is as follows:

(9) $$T_{ij}=1-z_{ij}$$

(10) $$O_{j}=\frac{w_{j}\times T_{ij}}{\sum_{i=1}^{n}\left(w_{j}\times T_{ij}\right)}\times 100\%$$

where $O_{j}$ represents the obstacle degree of the j th indicator on the safety state, which means the influence degree of each indicator or unit on the overall food security status. $z_{ij}$ is the normalized value of the single index, and $T_{ij}$ refers to the deviation degree of the indicator, which represents the difference between the actual indicator value and the optimal target value.

## 3. Results

### 3.1. Change Trend in China’s Food Security

**Figure 1:**
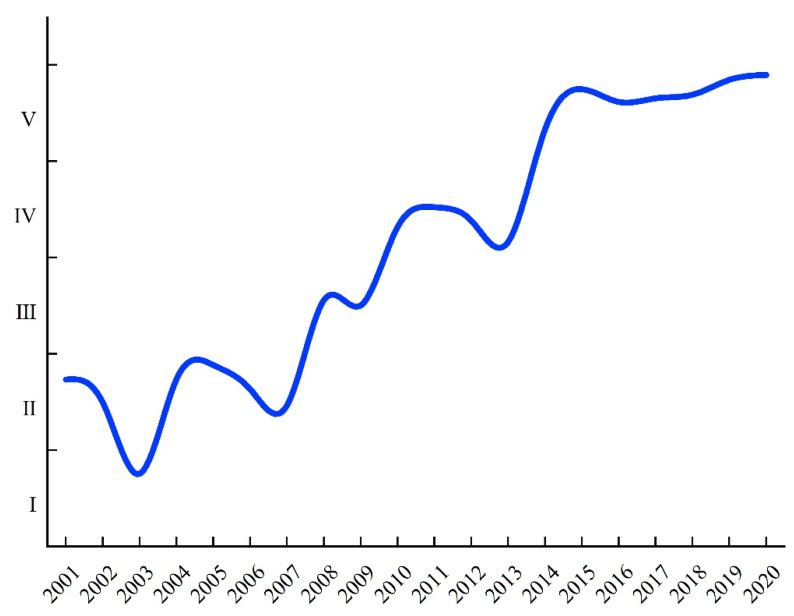
Trend map of China’s food security grades from 2001 to 2020.

A trend map of food security in China from 2001 to 2020 can be drawn according to the comprehensive correlation degree and evaluation grade (see Table A1 for more details). As shown in Figure 1, except for a state of relative insecurity in 2003, China’s overall food security displayed a substantial upward trend with fluctuations during this period. In 2003, the planting area of grain crops in China was less than 100 million hectares, accounting for 65.2% of the total sown area of crops, a record low level [38]. Another possible explanation for this might be that China’s food security is affected by international grain prices and the affordability of food for its residents. Affected by the significant reduction in food production in 2003, China’s food market prices rose for the first time in six years, and international food prices also increased.

After 2003, China welcomed 17 consecutive large harvests. There were adequate grain supplies and reserves and a stable grain market, which are indicators of increasing food security. However, the fluctuation in the comprehensive correlation degree curve of food security suggests that China’s food security cannot be guaranteed only by an increase in total grain output, as it is based on the joint effect of multiple factors. Therefore, it depends on maintaining the sustainability and stability of agricultural production based on steadily improving food production, prioritizing the protection of resources and the environment, strengthening agricultural infrastructure construction, improving agricultural production capacity and risk response capacity, and ensuring its long-term stability.

### 3.2. Factors Restricting China’s Food Security

**Figure 2:**
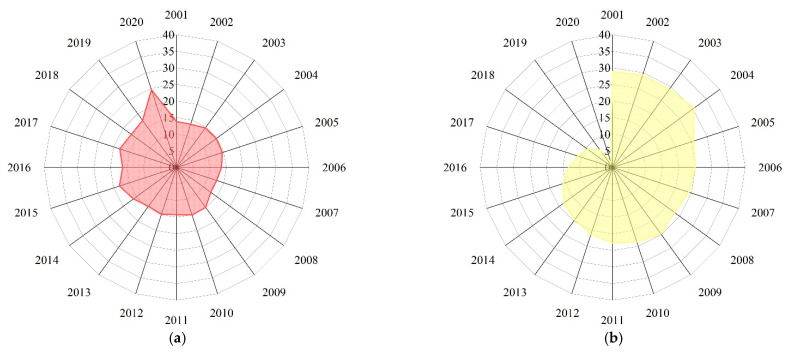

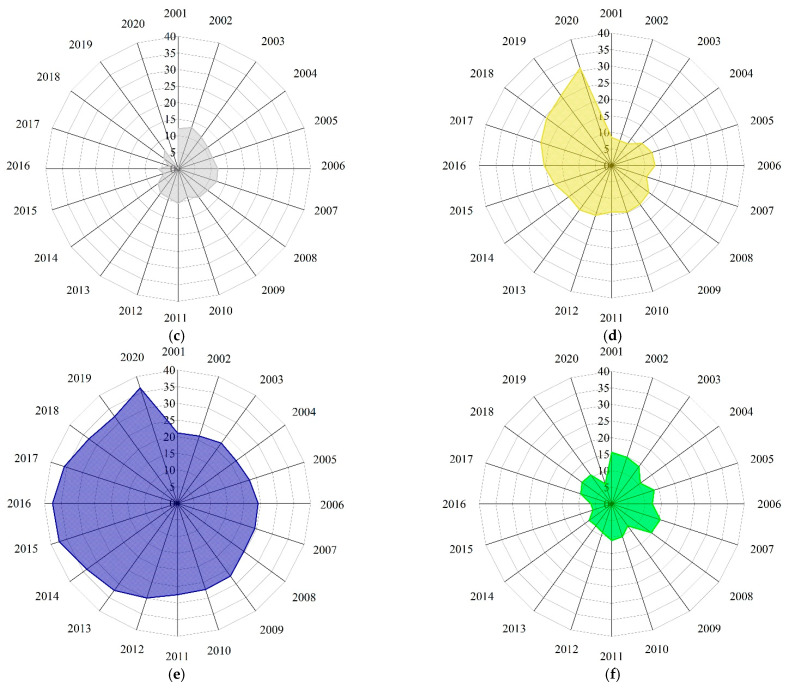
Radar chart of the obstacle degree of each unit from 2001 to 2020. (**a**) Availability, (**b**) distribution, (**c**) utilization, (**d**) vulnerability, (**e**) sustainability and (**f**) regulation.

**Figure 3:**
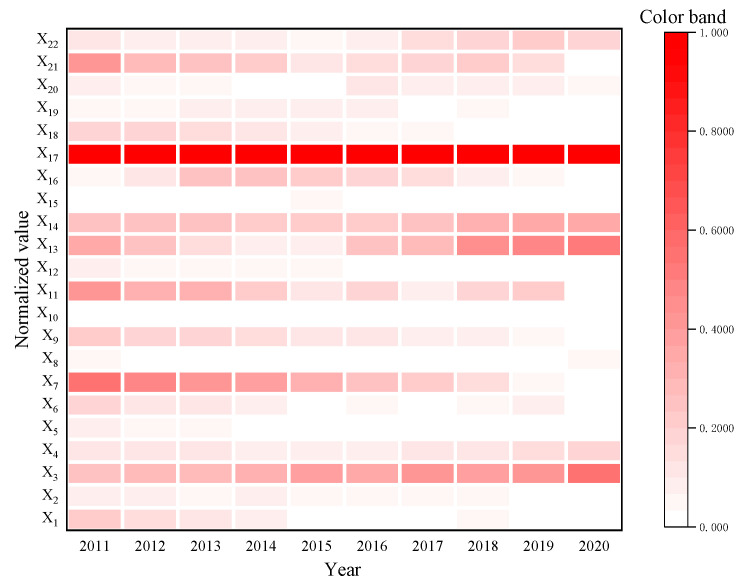
Heatmap of the obstacle degree of each index from 2011 to 2020.

Figure 2 reflects the change trend of the unit obstacle degrees for China’s food security: availability, distribution, utilization, vulnerability, sustainability, and regulation from 2001 to 2020 (see Table A2 for more details). Figure 3 shows the heatmap of each index after normalization of the obstacle degree from 2011 to 2020. Generally, the obstacle degrees of vulnerability and sustainability displayed a slow upward trend, while grain distribution as an obstacle exhibited a downward trend in volatility, and both utilization efficiency and government regulation displayed a small downward trend and then remained at a lower level.

It is generally accepted that adequate food availability is the ‘ballast stone’ of food security and a critical factor for responding effectively to international market shocks. During 2001–2019, overall food availability remained stable, and grain availability as an obstacle was approximately 15%, while the degree to which grain availability was an obstacle increased to 24.6% in 2020 (Figure 2a). As noted, China’s GY and TGP have been increasing, especially since 2003. Grain production has experienced seventeen years of consecutive production increases, and grain availability has generally been stable. However, the obstacle degree of food availability increased in 2020, and the GSR has also gradually emerged as an obstacle, mainly because net grain imports in 2020 were 30.3 million tons more than in 2019, an increase of 1.3% (in 2019, imported wheat, corn, and soybeans totaled 3.5, 4.8, and 88.5 million tons, respectively, compared to 8.4, 11.2, and 100.3 million tons in 2020) [39,40]. At present, China’s GSR exceeds 95% [41], which provides a solid foundation for ensuring China’s food security and promoting economic and social development as well as long-term national stability.

Grain distribution has always been the most important factor affecting China’s food security. Since entering the new century, China has substantially improved its transportation construction and infrastructure, the national economy has developed rapidly and stably, and grain distribution as an obstacle to China’s food security has decreased dramatically, from 28.9% in 2001 to 9.4% in 2020 (Figure 2b). After years of infrastructure construction, China’s transportation conditions have greatly improved, and railway and highway mileage are increasing, which play a positive role in ensuring China’s grain circulation. According to the National Bureau of Statistics of China, by the end of 2000, the first national trunk line of the Beijing and Shanghai Expressway was fully connected, Chinese mainland expressway mileage had reached 16,000 km, railway business mileage was 687,000 km, and the grain freight ton-kilometers was 101.3 billion ton-km. However, by the end of 2020, mainland Chinese highway mileage was 5.2 million kilometers, of which China’s 161,000 km of expressways ranked first in the world. The length of China’s railways in operation reached 146,300 km, also ranked first worldwide, with grain freight traffic at 75.7 million tons and grain freight ton-kilometers at 139.2 billion [40,42]. Meanwhile, China’s achievements in transportation development were the dominant factor effectively promoting rapid economic and social development and providing a solid guarantee that residents could obtain sufficient food for consumption. Since the onset of reform and opening up, China’s economy has developed rapidly. In 2000, China’s GDP exceeded RMB 10 trillion, with China becoming the world’s sixth-largest economy. In 2020, China’s GDP reached RMB 101 trillion, and the per capita gross national income was RMB 71,489, which was higher than the average level of middle-income countries, substantially improving the food availability capacity of urban and rural residents [40,42].

As shown in Figure 2d,e, the vulnerability of China’s food security experienced rapid growth from 8.4% to 31.0%, while the obstacle of sustainability experienced a slow upward trend from 21.2% to 36.4% between 2001 and 2020. With multifaceted changes in the global climate, the frequent occurrence of extreme natural disasters has strengthened the link between food security stability and extreme climate change. Furthermore, whether agriculture can achieve sustainable development depends on the sustainable supply of natural resources, the most important of which are water resources and cultivated land. On the one hand, coincident with the rapid development of contemporary society, the contradiction between agricultural water shortages and increasing demand for domestic water has become more prominent. Another limitation is that China’s rainwater resources are unevenly distributed, and there are large seasonal differences. On the other hand, at present, more than 116 million hectares are sown with grain, an increase of approximately 8.3 million hectares over 2000 [40]. Although the foundations of grain production have been strengthened, it is worth noting that the AFA has become the largest obstacle affecting food security in the past decade. The continuous decline in the quality of cultivated land is destroying farmland ecosystems and endangering human health, which significantly restricts China’s grain production ability.

Figure 2c,f show the obstacle degrees of utilization efficiency and government regulation restricting China’s food security during 2001–2020. It is also found that utilization efficiency and government regulation had little impact on food security, showing a small decline in fluctuations, indicating that the continuous increase in China’s food security is closely related to agricultural and rural support and government financial support. The popularization and application of agricultural science and technology have played a positive role, substantially reducing the grain loss rate at various stages, such as processing, storage, and circulation. Additionally, the Chinese government has instituted a series of national policies to support, benefit, and enrich agriculture and further alleviate poverty, resulting in noticeable improvements to nutrition and health.

Table 2 presents the primary obstacle indicators among the 22 indicators of China’s food security during 2011–2020. The table shows that the top five obstacle factors are mainly reflected in the sustainability and vulnerability of food security. Specifically, during 2011–2020, the largest limiting factor affecting food security was the AFA. This suggests that there are large differences in land quality grades across the country. When there is insufficient fertile cultivated land, soil fertility is improved by increasing the application of chemical fertilizer, which severely restricts sustainable agricultural production. Meanwhile, from 2011 to 2014, the status of grain distribution also affected China’s food security, and pDIR became the second-largest risk factor. However, since 2014, the GSR has gradually become the second-largest risk factor limiting the development of China’s food security. As discussed, this is because of the rapid increase in corn and soybean imports; China’s actual grain self-sufficiency rate has shown an overall downward trend, the structural contradiction of grain varieties in China has intensified, and the development of the agricultural structure has become unbalanced. In addition, the GRL and pSA have declined slowly, which indicates that China’s grain production is insufficiently stable and hinders food security development. In recent years, restricted by natural conditions, climate change, market conditions, and other factors, the stability of food production has also had a crucial impact on China’s food security.

## 4. Discussion

### 4.1. China’s Food Security

In this work, we use a scientific method to comprehensively and effectively evaluate China’s food security, which plays a crucial role in determining the central goals of China’s food security strategy and agricultural policy. China’s food security status increased gradually from 2001 to 2020 but exhibited a slight downward trend in 2003. These results indicate that China’s overall food security has been consistently improving. Our findings are supported by Wu et al. [43] and Yao et al. [44], who found that the grain sowing area decreased and the planting area of cash crops increased amid changes to China’s agricultural structure in 2003, resulting in a significant reduction in China’s total grain output.

Adequate grain availability is the fundamental factor affecting food security. The rapid development of agricultural science and technology does seem to steadily improve China’s comprehensive grain production capacity. However, China is the world’s largest grain importer and imports a large amount of grain every year [45]. Cereal has basically achieved full self-sufficiency, consistent with several populous developing countries, though lower than developed countries, but soybean self-sufficiency has declined significantly [29]. In 2020, 73.0% of China’s imported rice came from Vietnam, Pakistan, Thailand, and Cambodia. In addition to rice, the sources of wheat and corn are mainly concentrated in resource-rich countries or regions such as the United States, Brazil, Argentina, and the European Union [46]. China’s soybean dependence on foreign countries reached a very high level. Specifically, China’s soybean imports enjoyed rapid growth from 13.9 to 100.3 million tons between 2001 and 2020. The main soybean importers are Brazil, the United States, and Argentina, with Brazil accounting for 64% of the total [47,48]. Therefore, changes to agricultural layouts and grain planting structures should be further promoted; grain planting areas should be stabilized; and potatoes, beans, miscellaneous grains, and other crops should be developed according to local conditions.

Agricultural costs are still rising, and resource and environmental carrying capacity is surpassing its limit [49]. We speculate that due to China’s low agricultural production capacity and extensive agricultural management, the ecological environment was extremely fragile, which hindered the improvement in grain production capacity to a large extent. In 2019, the consumption of chemical fertilizers for agriculture exceeded 55 million tons, and the AFA of farm crops remained at 325 kg/ha, far higher than the global average (120 kg/ha). Other data show that in 2019, the amount of pesticide application was 8.7 kg/ha, 3.3 times the global average [50]. The data contribute a clear understanding that China’s agriculture remains extensive and that technical knowledge, economic efficiency, and economies of scale are low. Agricultural producers often adopt excessive and predatory production modes; use large amounts of chemicals, such as pesticides, chemical fertilizers, and herbicides; and discharge livestock and poultry waste and domestic pollutants indiscriminately, resulting in the degradation and desertification of cultivated land resources. Agricultural infrastructure is comparatively weak, and the capacity for disaster prevention and relief must be improved. Moreover, unpredictable weather has led to a sharp decline in grain production and residents’ incomes in vulnerable areas and a sharp increase in the risk of food insecurity [51,52]. China will find itself under considerable pressure to maintain steady grain production, while ensuring green development and sustainable resource use. Thus, agricultural producers should control the application of chemical fertilizers and pesticides according to soil fertility, continue to strengthen the treatment of agricultural nonpoint source pollution, and seek to achieve high-quality agricultural development.

The results demonstrate that the overall situation of China’s food security has been significantly improved but is not balanced in different dimensions. Our findings are in accord with recent studies indicating that large populations, climate change, and reduced resources are the prevailing challenges to current food security in most developing countries [12,13,53]. Pakistan, for example, faces significant future challenges to feed a growing population, given that the area of crop production is limited by the availability of fresh water for irrigation [54,55,56]. The international community has launched a wide range of discussions and cooperation in recent years to control greenhouse gas emissions [57]. To fulfil the international commitments of reducing carbon emissions, the Chinese government pledged to peak its growing carbon emissions by 2030 and a vision of achieving carbon neutrality by 2060 [58]. However, global major crops are sensitive to climate change, including changes in temperature, precipitation, and atmospheric carbon dioxide concentration [59,60,61]. The next 40 years could be the most critical period for ensuring China’s food security, which incorporates demographic, climate change, and resource shortage factors.

In view of severe food security challenges, China has a long way to go to facilitate the high-quality development of its grain industries and strengthen the food security guarantee. China will implement its national strategies for food security through sustainable farmland use and agricultural technology innovation to increase farmland productivity [62]. At present, China has carried out soil testing and formula fertilization; popularized the practice of returning straw to the field, green manure planting, the application of organic fertilizer, soil improvement, and other supporting technologies; and steadily improved the quality of cultivated land. China will continue to innovate in the seed industry, making breakthroughs in core technologies such as germplasm improvement and the creation, efficient cultivation, processing, and circulation of new crop varieties. China will enhance integrated technological innovation, breaking logjams in improving per unit area yield, crop quality, economic benefits, and the environment. At the same time, China intends to strengthen macroeconomic regulation and its support of agriculture, promote mechanization in agriculture, transform and upgrade the agricultural machinery industry, increase the grain supply, and improve grain quality through the application of agronomy and agrotechniques.

### 4.2. Limitations and Future Research Directions

Although this paper provides a more objective and practical food security evaluation system for China based on current circumstances, it suffers from several limitations and uncertainties. For one, the meaning of food security is far broader than the current index system reflects. In addition, because of data limitations, it was difficult for this study to provide a comprehensive analysis of food security. In future research, we will further analyze China’s food security according to the structural characteristics of grain crops to ensure that our results are more comprehensive.

## 5. Conclusions

In this study, we developed a new framework using six influencing units of food security (availability, distribution, utilization, vulnerability, sustainability, and regulation) to comprehensively measure China’s food security status in a changing world. We first constructed a more suitable and operational food security evaluation system for China based on current circumstances by adopting the EWM. Second, we examined China’s food security grade between 2001 and 2020 through the MEEM. Finally, the ODM was used to investigate the key obstacles restricting China’s food security. The conclusion of this study is based on the analysis of the influencing factors as a whole, and the results elucidate the steps that China should take to ensure its food security. The main conclusions are as follows.

(1) Except for a slight downward trend in 2003, China’s overall food security displayed a substantial upward trend during 2001–2020. The fluctuation in the comprehensive correlation degree trend map of food security suggests that China’s food security cannot be guaranteed only by an increase in total grain output but involves the joint effect of many factors. The next 40 years could be the most critical period for ensuring China’s food security, which incorporates demographic, climate change, and resource shortage factors. Therefore, to fully modernize China’s agriculture, the food security strategy must be consistent with stable agricultural production and a sustainable development strategy.
(2) According to the change trend of China’s food security, in terms of the obstacle degrees of the indicators between 2001 and 2020, the obstacle degree of grain distribution exhibited a strong downward trend, while vulnerability and sustainability displayed a slow upward trend. China is a country with a large population and few resources; thus, China’s per capita share of various resources is extremely small. With the shortage of cultivated land and water resources available for agriculture, issues related to resources and the environment have become a bottleneck restricting the strength and sustainability of food security in China.
(3) It is worth noting that AFA and GSR have gradually become obstacles to the development of China’s food security. The long-term use of pesticides and chemical fertilizers may have resulted in a shortage of agricultural resources, restricting the potential to expand agricultural reproduction. Moreover, the high degree of dependence on foreign countries for soybeans and the primary grain structural conflicts that such dependence involves represent a potential threat to China’s food security. Thus, China appears to be implementing its national strategies through sustainable farmland use and agricultural technology innovation to facilitate the high-quality development of its grain industries and strengthen food security.

## Figures and Tables

**Table 1:** Evaluation index system of China’s food security.

Index/Unit		Index Definition/Explanation
Availability (A)	Total grain production (TGP)/10^4^ ton	Total output of grain produced within a calendar year
	Grain yield per unit area (GY)/(kg/hm^2^)	Productive capacity of cultivated land within a region
	Grain self-sufficiency rate (GSR)/%	Grain production/(grain production + imports − exports)
	Planting structure of grain crops (PSG)/%	Proportion of the total sown area of grain crops to farm crops
Distribution (D)	Per capita grain availability (pGA)/(kg/person)	Total grain production/total population
	Per capital daily total intake (pDTI)/(kcal/day/person)	(Total grain production + net grain import) × grain comprehensive calories/(total population × 365)
	Per capita disposable income of residents (pDIR)/RMB	Purchasing power of residents
	Grain price volatility index (GPVI)/preceding year = 100	Grain retail price fluctuant trend and degree
	Density of highways and railways (DHR)/10^4^ km	Effectiveness of transportation in grain distribution and access
Utilization (U)	Prevalence of undernourishment (PU)/%	Proportion of the population failing to meet dietary needs for a healthy life to the total population
	Loss rate (LR)/%	Utilization efficiency of five main crops in each stage (rice, wheat, corn, beans, and tubers)
	Poverty incidence (PI)/%	Impoverished population/total population
Vulnerability (V)	Grain reserve level (GRL)/%	Stock variation of state-owned grain enterprise
	Per capita sown area of grain crops (pSA)/(hm^2^/person)	Sown area of grain crops/total population
	Grain production fluctuation coefficient (GFC)/%	Fluctuation range of grain production
	Occurrence of natural disasters (OND)/case	Number of natural disasters in a year
Sustainability (S)	Amount of fertilizer application per unit sown area (AFA)/(kg/hm^2^)	Consumption of chemical fertilizer/sown area of grain crops
	Effective irrigation area (EIA)/10^3^ hm^2^	Area of cultivated land under normal irrigation for agricultural production
	Area affected of grain crops (AAG)/10^3^ hm^2^	Extent of grain production affected by disasters
	Level of agricultural machinery (LAM)/10^4^ kW	Effect of science and technology support on agricultural sustainable development
Regulation (R)	Per capita level of government expenditure for supporting rural production (pSRP)/(RMB/person)	Government expenditure for supporting agricultural development
	Proportion of government expenditure for supporting agriculture to agricultural output value (PAGE)/%	Government expenditure for supporting agriculture/gross output value of agriculture

**Table 2:** Top five obstacle indicators of China’s food security during 2011–2020.

Year	Option	Indicator Order				
		1	2	3	4	5
2011	Indicator	AFA	pDIR	LR	pSRP	GRL
	Value	20.0	11.1	8.6	8.4	7.1
2012	Indicator	AFA	pDIR	LR	pSRP	GSR
	Value	23.1	11.1	7.6	7.0	6.5
2013	Indicator	AFA	pDIR	LR	GSR	OND
	Value	24.8	10.6	7.6	7.3	6.5
2014	Indicator	AFA	pDIR	GSR	OND	pSA
	Value	27.6	10.2	9.0	6.8	6.3
2015	Indicator	AFA	GSR	pDIR	pSA	OND
	Value	32.3	12.4	10.3	7.1	6.7
2016	Indicator	AFA	GSR	pDIR	GRL	pSA
	Value	30.1	11.0	8.0	7.4	6.9
2017	Indicator	AFA	GSR	GRL	pSA	pDIR
	Value	31.1	12.8	9.2	8.2	6.3
2018	Indicator	AFA	GRL	GSR	pSA	pSRP
	Value	28.5	12.5	11.2	8.6	5.7
2019	Indicator	AFA	GRL	GSR	pSA	LR
	Value	29.5	14.2	12.1	10.3	6.8
2020	Indicator	AFA	GSR	GRL	pSA	PAGE
	Value	34.5	18.5	18.2	12.6	6.3

## Data Availability

Not applicable.

## Appendix A

### Appendix A.1. Comprehensive Correlation Degree and Evaluation Grade of China’s Food Security

The comprehensive correlation degree and evaluation grade of China’s food security can be calculated by Formulas (7) and (8), as presented in Table A1.

**Table A1:** Comprehensive correlation degrees and grades of China’s food security during 2001–2020.

	I	II	III	IV	V	$\mathrm{MAX}K\left(t\right)$	Grade
$K\left(t_{2001}\right)$	−0.1022	−0.0538	−0.3123	−0.4865	−0.6292	−0.0538	I
$K\left(t_{2002}\right)$	−0.1294	−0.1018	−0.3081	−0.5038	−0.6053	−0.1018	II
$K\left(t_{2003}\right)$	−0.0492	−0.2155	−0.4027	−0.5267	−0.6438	−0.0492	I
$K\left(t_{2004}\right)$	−0.1911	−0.0528	−0.3319	−0.4765	−0.5636	−0.0528	II
$K\left(t_{2005}\right)$	−0.2602	−0.0235	−0.1852	−0.3791	−0.5464	−0.0235	II
$K\left(t_{2006}\right)$	−0.2624	−0.0736	−0.1954	−0.3573	−0.5375	−0.0736	II
$K\left(t_{2007}\right)$	−0.2447	−0.1076	−0.1733	−0.3578	−0.5293	−0.1076	II
$K\left(t_{2008}\right)$	−0.3168	−0.1120	−0.0883	−0.2865	−0.4777	−0.0883	III
$K\left(t_{2009}\right)$	−0.3801	−0.2333	−0.0995	−0.2558	−0.4033	−0.0995	III
$K\left(t_{2010}\right)$	−0.3839	−0.2974	−0.1394	−0.1364	−0.3817	−0.1364	IV
$K\left(t_{2011}\right)$	−0.4250	−0.3730	−0.2383	−0.0957	−0.3350	−0.0957	IV
$K\left(t_{2012}\right)$	−0.4903	−0.4135	−0.2854	−0.1235	−0.2369	−0.1235	IV
$K\left(t_{2013}\right)$	−0.5094	−0.4833	−0.3179	−0.1683	−0.1892	−0.1683	IV
$K\left(t_{2014}\right)$	−0.5525	−0.5361	−0.4267	−0.2038	−0.1336	−0.1336	V
$K\left(t_{2015}\right)$	−0.6077	−0.6103	−0.5360	−0.3333	−0.0505	−0.0505	V
$K\left(t_{2016}\right)$	−0.6048	−0.5555	−0.5187	−0.2709	−0.0770	−0.0770	V
$K\left(t_{2017}\right)$	−0.6175	−0.5869	−0.5745	−0.3562	−0.0696	−0.0696	V
$K\left(t_{2018}\right)$	−0.6168	−0.5891	−0.5578	−0.3209	−0.0619	−0.0619	V
$K\left(t_{2019}\right)$	−0.6655	−0.5922	−0.5992	−0.4118	−0.0313	−0.0313	V
$K\left(t_{2020}\right)$	−0.7123	−0.6720	−0.7164	−0.6238	−0.0209	−0.0209	V

### Appendix A.2. Obstacle Degree of Six Units Relating to China’s Food Security

The obstacle degree of each unit restricting the development of China’s food security during 2001–2020 is identified based on the ODM, as presented in Table A2.

**Table A2:** Obstacle degree of six units relating to China’s food security during 2001–2020.

Year	Availability	Distribution	Utilization	Vulnerability	Sustainability	Regulation
2001	13.82	28.90	12.11	8.43	21.15	15.59
2002	13.69	29.26	13.15	7.87	21.25	14.78
2003	14.70	29.04	11.86	8.12	22.38	13.90
2004	14.82	30.44	11.01	11.31	21.78	10.65
2005	14.54	25.70	10.86	12.74	22.73	13.43
2006	13.55	24.97	11.96	13.05	24.24	12.23
2007	12.71	24.18	12.23	11.05	24.45	15.37
2008	12.76	23.09	11.00	13.79	24.63	14.73
2009	14.89	24.78	10.68	14.43	27.06	8.16
2010	15.09	23.59	8.90	14.82	27.25	10.36
2011	14.22	22.83	10.42	14.01	27.49	11.03
2012	14.84	21.17	9.20	15.82	29.93	9.04
2013	14.49	19.49	9.04	16.52	32.37	8.09
2014	16.13	18.21	7.51	16.01	33.74	8.40
2015	18.17	15.77	4.57	18.12	37.40	5.98
2016	16.33	13.32	6.04	20.45	37.58	6.28
2017	18.06	10.98	2.89	22.46	35.77	9.83
2018	16.94	9.43	5.29	24.32	32.94	11.07
2019	17.49	6.50	6.99	26.07	32.15	10.80
2020	24.63	1.69	2.53E-03	30.95	36.42	6.32
